# The Latest Option: Nivolumab and Relatlimab in Advanced Melanoma

**DOI:** 10.1007/s11912-023-01406-4

**Published:** 2023-04-01

**Authors:** Lea Jessica Albrecht, Elisabeth Livingstone, Lisa Zimmer, Dirk Schadendorf

**Affiliations:** 1 Department of Dermatology, University Hospital Essen, Hufelandstrasse 55, 45147 Essen, Germany; 2 The German Cancer Consortium (DKTK), Essen, Germany; 3 Comprehensive Cancer Center (Westdeutsches Tumorzentrum), University Hospital Essen, Essen, Germany; 4 NCT-West, Campus Essen and University Alliance Ruhr, Research Center One Health, University Duisburg-Essen, Essen, Germany

**Keywords:** Dual checkpoint inhibition, Relatlimab plus nivolumab, LAG-3 blocking antibody, Melanoma, T-cell exhaustion, Overcoming anti-PD-1 resistance

## Abstract

**Purpose of Review:**

Dual immune checkpoint inhibition with ipilimumab plus nivolumab is currently the most effective, but also by far the most toxic treatment for advanced melanoma. Therefore, other combination partners that also lead to high and long-lasting responses but cause fewer adverse events were explored.

**Recent Findings:**

Relatlimab, a LAG-3 blocking antibody, was investigated in combination with nivolumab in a phase 2/3 randomized double-blind trial (RELATIVITY-047) and could demonstrate significantly improved progression-free survival in treatment-naive advanced melanoma patients compared with nivolumab monotherapy. While the safety profile is more favorable than that of ipilimumab plus nivolumab, no significant survival benefit has yet been demonstrated with the new combination over nivolumab monotherapy.

**Summary:**

The approval of relatlimab plus nivolumab by both the Food and Drug Administration and the European Medicines Agency expands the arsenal of treatment options for melanoma but raises new questions in clinical practice and a re-evaluation of currently established treatment standards and sequences.

## Introduction

The development of immune checkpoint inhibitors has fundamentally revolutionized the therapeutic landscape and has led to a marked improvement in survival outcomes in patients with advanced melanoma by enabling profound and durable responses [1]. Based on the results of two randomized, double-blind trials (CheckMate 069, CheckMate 067) combined immunotherapy with anti-cytotoxic T lymphocyte antigen-4 antibody (anti-CTLA-4) ipilimumab plus anti-programmed cell death protein 1 antibody (anti-PD-1) nivolumab was the first dual checkpoint blockade approved by the Food and Drug Administration (FDA) for the treatment of metastatic melanoma [2, 3]. Since dual immunotherapy has demonstrated its clear superiority over anti-CTLA-4 monotherapy in both progression-free survival (PFS) and overall survival (OS), it is nowadays the standard of care for first-line treatment [3]. However, ipilimumab plus nivolumab is associated with a high risk of toxicity inducing a range of immune-related adverse events, and approximately 40% of patients discontinue treatment prematurely [4]. Moreover, a relevant subset of patients shows primary non-response or develops disease progression after a period of response [5, 6], thus many studies focus on overcoming resistance. The molecular mechanisms of resistance have not been fully elucidated to date. Since the expression of distinct immune checkpoint receptors could be a possible explanation, the identification of new checkpoint targets represents an appealing approach. Lymphocyte-activation gene 3 (LAG-3), a cell surface molecule on immune cells, which negatively regulates immune responses and is often co-expressed with PD-1 [7], represents one of the latest immune checkpoint receptors. In the phase 2/3 randomized double-blind study (RELATIVITY-047), relatlimab, the first-in-class LAG-3 inhibiting antibody (anti-LAG-3), demonstrated its efficacy, safety, and superiority over nivolumab monotherapy in a fixed-dose combination with nivolumab and represents the first dual checkpoint blockade to be approved along with ipilimumab and nivolumab [8••, 9]. Herein, we provide an overview of the latest therapeutic option of dual checkpoint blockade with relatlimab and nivolumab in melanoma and highlight its antitumor activity in different stages and patient populations. Moreover, we summarize the current state of clinical investigation of LAG-3 targeting molecules in melanoma and discuss the position of dual checkpoint inhibition with anti-LAG-3 plus anti-PD-1 antibodies in the arsenal of current melanoma therapies.

## Molecular Insights of LAG-3 Function

Immune checkpoints are membrane-bound receptors expressed by immune or tumor cells that lead to positive or negative regulation of the immune response [10]. Inhibitory receptors, such as CTLA-4 and PD-1, are physiologically upregulated during activation, expansion, and differentiation of naive T cells, especially during permanent antigen presentation, to maintain self-tolerance, i.e., suppress autoimmunity and prevent tissue damage, and thus contribute to immune escape and T cell exhaustion in carcinogenesis [11, 12]. Immune checkpoint inhibitors exert their effects by antagonizing the interaction between receptors and their ligands, thus counteracting immune exhaustion by activating a tumor-specific immune response [10, 13]. The LAG-3 gene (also known as CD223), first identified in 1990 [14] is located on chromosome 12 (12p13.32), like the coreceptor CD4, and encodes a 70 KDa single-pass transmembrane glycoprotein consisting of 498 amino acids [15]. It belongs to the Ig superfamily and contains an extracellular, a transmembrane and an intracellular region with four extracellular immunoglobulin-like superfamily regions (D1-D4) with one variable (type V) and three constant (type C) Ig-like domains [15–17]. LAG-3 is expressed on the surface of CD4 ^+^ and CD8 ^+^ T lymphocytes and inhibits the tumor immunological microenvironment by negatively affecting T cell proliferation and inducing T cell exhaustion [18–20]. It has been shown to be frequently co-expressed with PD-1, whereby high LAG-3 expression is found primarily in tumor-infiltrating T cells [7, 21]. Additionally, LAG-3 can be detected on other cell populations such as natural killer cells, NK T cells, regulatory T cells, dendritic cells, and activated B cells, although it is not clear whether expression on these cell populations contributes to antitumor immunity [18, 22–24]. Major histocompatibility complex class II (MHC II) molecules, which are highly expressed in cutaneous melanomas [25], represent the canonical ligands of LAG-3, as they do for CD4, however, the proline-rich D1 domain allows LAG-3 to bind with higher affinity to MHC II than to CD4 [26–28]. In addition to MHC II molecules, other ligands of LAG-3 have been described previously [29]. Based on its molecular function, constitutive LAG-3 expression may limit the antitumor effect of PD-1 blockade in treatment-naive patients, and combined checkpoint inhibition might improve response and increase its durability. Moreover, adaptive upregulation of LAG-3 expression may result in treatment resistance and tumor progression in patients receiving anti-PD-1 therapy, and anti-LAG-3 in combination with nivolumab could potentially restore T cell activation and tumor response.

## New Combined Checkpoint Inhibition with Anti-LAG-3 Antibody Relatlimab Plus Anti-PD-1 Antibody Nivolumab in Advanced Melanoma

Relatlimab is a first-in-class human IgG4-LAG-3 blocking antibody that, in combination with nivolumab, is the third immune checkpoint inhibitor to receive approval for the treatment of patients with advanced melanoma. Relatlimab plus nivolumab is a fixed-dose combination immunotherapy for the treatment of various advanced-stage cancer entities and received FDA approval in March 2022 for the treatment of advanced melanoma in adult patients and children ≥ 12 years of age weighing at least 40 kg [30]. On 09/15/2022, the European Medicines Agency (EMA) issued marketing authorization for relatlimab plus nivolumab throughout the European Union for patients 12 years of age and older with unresectable or metastatic melanoma with programmed cell death ligand 1 (PD-L1) expression less than 1% [31]. The approval based on the results of a global, double-blind, randomized phase 2/3 study (RELATIVITY-047), demonstrating the superiority in PFS of combined PD-1/LAG-3 inhibition with relatlimab plus nivolumab compared to nivolumab alone in patients with untreated metastatic or unresectable melanoma [8••]. Previously, the phase 1/2 dose escalation and cohort expansion study (RELATIVITY-020; NCT01968109) which also included patients with advanced melanoma who had failed or exhibited disease progression to anti-PD-1 therapy was able to prove favorable tolerability and long-term response to relatlimab plus nivolumab with an overall response rate (ORR) of 16% and disease control rate (DCR) of 45% [32].

First-line treatment with relatlimab plus nivolumab resulted in improved and more than doubled PFS (10.1 vs 4.6 months) compared with nivolumab monotherapy after a median follow-up of 13.2 months, with a nearly 12% difference in PFS at 12-month follow-up and an overall risk reduction of disease progression of 25% compared to nivolumab monotherapy (hazard ratio (HR) 0.75; 95% confidence interval (CI), 0.62 to 0.92; *P* = 0.006). In an updated report at a median follow-up time of 19.3 months, no major change in PFS between the two treatment groups (10.2 vs 4.6 months; HR 0.78; 95% CI 0.64–0.94) has been observed. Moreover, treatment with nivolumab plus relatlimab resulted in numerically improved ORR (ORR: complete response (CR) + partial response (PR)) (43.1 vs 32.6%) and DCR (DCR: CR + PR + stable disease (SD)) (62.8 vs 50.7%) compared to nivolumab monotherapy. Median OS has not yet been reached in patients treated with relatlimab plus nivolumab, as opposed to nivolumab monotherapy (34.1 months, 95%CI 25.2 - not reached (NR)), yielding a 20% risk reduction of death to date (HR 0.8; 95%CI 0.64–1.01; *P* = 0.0593) [33••]. Although cross-trial comparisons should be made with caution, and survival data from the Checkmate 067 trial were investigator-assessed and not by a blinded independent central review (BICR) as in the RELATIVITY047 trial, ipilimumab plus nivolumab and relatlimab plus nivolumab show comparable efficacy data with similar PFS rates (2-year PFS; 38.5 vs 43%) and OS rate (2-year OS; 63.7 vs 64.0%) (Table 1) 2, 4, 8••, 34.

**Table 1:** Comparison of efficacy data of dual checkpoint inhibition [2, 4, 8••, 34]

		RELATIVITY-047^1^ *(Assessment by BICR)*		Checkmate 067^2^ *(Assessment by investigator)*	
		Relatlimab /Nivolumab	Nivolumab	Ipilimumab /Nivolumab	Nivolumab
ORR %		43	33	58	44
Median PFS months (95% CI)		10.2 (6.5–14.8)	4.6 (3.48–6.44)	11.5 (8.7–19.3)	6.9 (5.1–9.7)
HR (95% CI)*		0.78 (0.64–0.94)		0.78 (0.64–0.96)	
Median OS months (95% CI)		NR (34.2-NR)	34.10 (25.23-NR)	72.1^3^ (38.2-NR)	37.6 (29.1-NR)
HR (95% CI)*		0.80 (0.64–1.01)		0.85 (0.68–1.07)**	
1-year	**PFS %** **(95% CI)**	48.0 (42.5–53.4)	36.9 (31.7–42.1)	50.0^4^ (44.0–55.0)	43.0^4^ 37.0–49.0)
	**OS %** **(95% CI)**	77.0 (72.2–81.1)	71.6 (66.6–76.0)	73.0^4^ (68.0–78.0)	74.0^4^ (69.0–79.0)
2-years	**PFS %** **(95% CI)**	38.5 (32.7–44.2)	29.0 (23.8–34.4)	43.0^4^ (37.0–48.0)	37.0^4^ (31.0–43.0)
	**OS %** **(95% CI)**	63.7 (58.1–68.7)	58.3 (52.7–63.4)	64.0^5^ (59.0–69.0)	59.0^5^ (53.0–64.0)

BICR: blinded independent central review; ORR: overall response rate; PFS: progression-free survival, HR: hazard ratio; OS: overall survival; NR: not reached; CI: confidence interval; *in comparison with nivolumab mono; **descriptive analysis; 1: median follow-up time: 19.3 months; 2: median follow-up time: 36 months; 3: minimum follow-up time 77 months; 4: minimum follow-up time 12.2–12.5 months; 5: minimum follow-up 28 months

Furthermore, treatment with relatlimab plus nivolumab showed superiority to monotherapy across all key subgroups. Dual checkpoint inhibition with relatlimab plus nivolumab resulted in prolonged PFS regardless of LAG-3 status, LAG-3 expression ≥ 1% was associated with superior PFS in both treatment arms. In contrast, patients with PD-L1 expression ≥ 1% did not benefit more from combination therapy (median PFS 15.7 vs 14.7 months). PFS was higher in both treatment groups when PD-L1 expression was ≥ 1%. However, in patients with low PD-L1 expression (< 1%), dual checkpoint inhibition resulted in longer PFS compared with nivolumab monotherapy (6.4 vs 2.9 months). Moreover, the benefit of relatlimab plus nivolumab was shown to be independent of v-raf murine sarcoma viral oncogene homolog B1 (BRAF*)* mutational status (median PFS in *BRAF* V600 and *BRAF* wild-type: 10.1 vs 4.6 months). Even with prognostically unfavorable factors such as increased LDH levels, increased tumor burden, or increasing American Joint Committee on Cancer (AJCC) M-stage, which are generally associated with shorter PFS, combined immunotherapy resulted in a better outcome independent of key prognostic factors and demographic data such as age and gender. The analysis of an early on-treatment biopsy in the phase 2 study showed an association between the immune-related pathological response and the radiological response at four weeks follow-up, whereby the highest major pathological response (≤ 10% residual viable tumor) rate was recorded under treatment with relatlimab plus nivolumab, thus validating the clinical benefit of combined immunotherapy at the pathological level. Thus, early-on treatment biopsy may serve as a biomarker of treatment response in advanced melanoma [35].

Dual therapy with relatlimab plus nivolumab showed manageable tolerability with an acceptable safety profile without new or unexpected safety signals (Table 2). The frequency of grade 3/4 treatment-related adverse events was higher with combination therapy (21.1 vs 11.1%), and more patients discontinued treatment due to adverse events (9.0 vs 3.6%) [33••]. The most common grade 3 or 4 treatment-related adverse events in the relatlimab-nivolumab group included elevated lipase, alanine aminotransferase, aspartate aminotransferase, and fatigue. Hypothyroidism, thyroiditis, rash, and diarrhea or colitis were the most common immune-mediated adverse events. Myocarditis was slightly more frequent under combination therapy compared to monotherapy. Overall, there was a more than half reduced risk of treatment-related adverse events grade 3/4 adverse events with the new combination compared with ipilimumab plus nivolumab (21 vs 59%) [2, 8••].

**Table 2:** Summary of adverse events of RELATIVITY-047 [8••]. *Adapted from Tawbi et al., N Engl J Med. 2022*

Adverse Event	Relatlimab-Nivolumab (*N* = 359)		Nivolumab (*N* = 355)	
	Any grade	Grade 3 or 4	Any grade	Grade 3 or 4
*number of events (%)*				
Any adverse event	345 (97.2)	143 (40.3)	339 (94.4)	120 (33.4)
Treatment-related adverse event	288 (81.1)	67 (18.9)	251 (69.9)	35 (9.7)
Led to discontinuation of treatment	52 (14.6)	30 (8.5)	24 (6.7)	11 (3.1)
*Treatment-related adverse event in* ≥ *10% of patients in the relatlimab–nivolumab group*				
Pruritus	83 (23.4)	0	57 (15.9)	2 (0.6)
Fatigue	82 (23.1)	4 (1.1)	46 (12.8)	1 (0.3)
Rash	55 (15.5)	3 (0.8)	43 (12.0)	2 (0.6)
Arthralgia	51 (14.4)	3 (0.8)	26 (7.2)	1 (0.3)
Hypothyroidism	51 (14.4)	0	43 (12.0)	0
Diarrhea	48 (13.5)	3 (0.8)	33 (9.2)	2 (0.6)
Vitiligo	37 (10.4)	0	35 (9.7)	0
*Immune-mediated adverse events*				
Hypothyroidism or thyroiditis	64 (18.0)	0	50 (13.9)	0
Rash	33 (9.3)	2 (0.6)	24 (6.7)	5 (1.4)
Diarrhea or colitis	24 (6.8)	4 (1.1)	11 (3.1)	5 (1.4)
Hyperthyroidism	22 (6.2)	0	24 (6.7)	0
Hepatitis	20 (5.6)	14 (3.9)	9 (2.5)	4 (1.1)
Adrenal insufficiency	15 (4.2)	5 (1.4)	3 (0.8)	0
Pneumonitis	13 (3.7)	2 (0.6)	6 (1.7)	2 (0.6)
Hypophysitis	9 (2.5)	1 (0.3)	3 (0.8)	1 (0.3)
Nephritis and renal dysfunction	7 (2.0)	4 (1.1)	5 (1.4)	4 (1.1)

Although the clinical benefit of LAG-3 inhibition with relatlimab and its establishment as a third immune checkpoint inhibitor has been clearly demonstrated, further investigation and studies are needed to understand the efficacy of relatlimab plus nivolumab in patient populations that are often excluded from clinical trials, such as patients with active or untreated brain metastases or with rare melanoma subtypes.

## Targeting LAG-3 in Uveal Melanoma

Uveal melanoma represents a rare subtype of melanoma but is the most common intraocular malignancy [36]. Although the diagnosis is usually made at an early stage and enucleation or brachytherapy provides effective local therapeutic control, approximately 50% of patients develop metastases, primarily to the liver [37, 38]. In contrast to cutaneous melanomas, uveal melanomas show unsatisfactory response rates to immune checkpoint inhibitors [39–41]. Only 3.6% of patients responded to anti-PD-1-based monotherapy with a median PFS of 2.6 months and OS of 7.6 months. Slightly better response rates of 15 to 18% were achieved with dual immune checkpoint blockade with ipilimumab and nivolumab, however, these rates are far below those achieved in cutaneous melanomas [42, 43]. As previously discussed, the expression of additional immune checkpoint receptors such as LAG-3 or distinct expression levels of these immune checkpoints could also explain the unfavorable response rates to anti-CTLA-4 and anti-PD-1 therapies in uveal melanoma. Analysis of The Cancer Genome Atlas (TCGA) datasets proved the presence of LAG-3 in uveal melanoma and its association with an increased rate of metastasis [44]. Increased LAG-3 expression correlated positively with high-risk factors such as epithelioid/mixed cell type and BAP1 loss, and high expression of both LAG-3 and its ligands was associated with unfavorable survival rates. Further analyses identified that the expression levels of checkpoint inhibitors of CD8 ^+^ T cells of the tumor microenvironment varied, showing high expression of LAG-3 and low expression of CTLA-4 and PD-1, thus identifying LAG-3 as the predominant checkpoint inhibitor [45], suggesting that dual immune checkpoint blockade with anti-LAG-3 may be the preferred treatment option in uveal melanoma [39]. In order to test this hypothesis, enrollment is currently in progress in an open-label, single arm, single site, investigator-initiated phase 2 study (CA224-094) evaluating the efficacy and safety of nivolumab in combination with relatlimab in patients with therapy-naive advanced uveal melanoma (NCT04552223) [46].

## (Neo-) Adjuvant Approaches with Relatlimab Plus Nivolumab

Anti-PD-1 antibodies have demonstrated their clinical benefit also as adjuvant treatment and have become part of the clinical routine in the management of fully resectable high-risk stage IIB-IV melanoma patients. Given the positive data of relatlimab plus nivolumab compared to anti-PD-1 monotherapy in metastatic and non-resectable stages, the question arises whether dual checkpoint inhibition will also outperform anti-PD-1 monotherapy in the adjuvant setting. Currently, a phase 3 study investigates the efficacy and tolerability of adjuvant therapy with relatlimab plus nivolumab compared to nivolumab monotherapy in patients with fully resected stage III/IV cutaneous melanoma (NCT05002569). Recruitment is expected to be reached by February 28^th^, 2023.

Neoadjuvant therapy approaches have been able to achieve significantly more robust immune responses to immune checkpoint inhibitors due to the intact tumor microenvironment compared to adjuvant therapy regimens [47], thus bringing them into the focus of current research. Early initiation of therapy potentially leads to eradication of micrometastases [48], reduction of tumor burden lowers surgical morbidity, and the ability to personalize adjuvant therapy based on pathologic response are further advantages in favor of neoadjuvant approaches. Patients who achieve a pathologic complete response with neoadjuvant therapy show significantly improved relapse-free survival with both targeted and immunotherapy-based therapeutic approaches [49]. In contrast to targeted therapy, the clinical benefit of neoadjuvant immunotherapy appears to be warranted with any pathologic response (pathological complete response (pCR), near pCR, pathological partial response (pPR)) [49]. Neoadjuvant therapeutic approaches based on dual immune checkpoint inhibition with ipilimumab and nivolumab result in more favorable response rates compared with anti-PD-1 monotherapy, however, they are associated with increased toxicity [50–52]. Based on the superiority of relatlimab plus nivolumab over monotherapy in advanced-stage melanoma and the improved outcome of neoadjuvant therapy approaches with dual checkpoint inhibition with ipilimumab plus nivolumab, it is hypothesized that combined immunotherapy with relatlimab plus nivolumab may also show its clinical benefit in the neoadjuvant setting. In a small phase 2 trial, patients with resectable stage IIIB/C or IV melanoma received two doses of relatlimab plus nivolumab 160/480 mg neoadjuvantly at 4-week intervals followed by adjuvant relatlimab plus nivolumab to complete one year [53]. Surgical resection was performed at week 9 followed by adjuvant therapy continuation. An overall radiographic response rate of 57% was recorded in a total of 30 included patients. In 29 patients who received surgical resection, a pCR rate of 59% and a near pathological response rate of 7% were observed. A major pathological response (pCR + near pCR: 66%) was associated with improved relapse-free survival, and radiologic evaluation of treatment response seems to underestimate the pathological response rate as has also been seen in other neoadjuvant trials. Moreover, neoadjuvant treatment did not show any treatment-related grade 3/4 adverse events or surgery delay. The adverse event rate of grade 3 side effects amounted to 26% in the adjuvant setting comparable to the adverse event rate in patients treated with relatlimab plus nivolumab in the advanced stage [53]. With a median follow-up time of 16.2 months, neoadjuvant treatment with relatlimab plus nivolumab was able to demonstrate high response rates and showed improved relapse-free survival without new safety signals. Notably, the efficacy appears to be comparable to that of neoadjuvant ipilimumab plus nivolumab but with significantly reduced toxicity and more favorable tolerability, though the number of treated patients was low [53]. Currently, many clinical trials are ongoing worldwide to evaluate the clinical benefit of relatlimab in different stages and conditions of melanoma (Table 3A).

**Table 3:** Ongoing clinical trials investigating anti-LAG3 molecules in melanoma

A	Clinical trials investigating relatlimab in melanoma				
Condition	Title			Trail ID	Status on ClinicalTrials.gov (Accessed on 27 August 2022)
Metastatic uveal melanoma	A phase 2 study of nivolumab + BMS-986016 (relatlimab) in patients with metastatic uveal melanoma			NCT04552223	Recruiting
Unresectable or metastatic melanoma	A phase 2 study of anti-PD1 monoclonal antibody (nivolumab, BMS-936558) administered in combination with anti-LAG3 monoclonal antibody (relatlimab, BMS-986016) in patients with metastatic melanoma naive to prior immunotherapy in the metastatic setting			NCT03743766	Recruiting
Unresectable or metastatic melanoma	A phase 1/2a study to evaluate the safety, tolerability, and efficacy of relatlimab administered in combination with ipilimumab or ipilimumab alone in participants with unresectable or metastatic melanoma who have progressed on anti-PD-1 therapy			NCT03978611	Recruiting
Completely resected stage III/IV melanoma	A phase 3, randomized, double-blind study of adjuvant immunotherapy wit relatlimab and nivolumab fixed-dose combination versus nivolumab monotherapy after complete resection of stage III-IV melanoma			NCT05002569	Recruiting
Stage II melanoma	A phase 2, open label, single arm, clinical trial of neoadjuvant relatlimab and nivolumab in high risk, clinical stage II cutaneous melanoma			NCT05418972	Not yet recruiting
B	Clinical trials investigating new anti-LAG3 molecules in advanced malignancies including melanoma				
Agent	Class	Condition	Phase	Trail ID	Status on ClinicalTrials.gov (Accessed on 27 August 2022)
REGN3767 Cemiplimab	Anti-LAG-3 monoclonal antibody Anti-PD-1 monoclonal antibody	Advanced malignancies	1	NCT03005782	Recruiting
RO7247669 (RG6139)	Anti-PD1-LAG-3 bispecific antibody	Solid tumors Metastatic melanoma Non-small cell lung cancer Esophageal squamous cell carcinoma	1	NCT04140500	Recruiting
IMP321 Avelumab	LAG-3 Ig Fusion Protein Anti-PD-L1 monoclonal antibody	Solid Tumors Peritoneal Carcinomatosis	1	NCT03252938	Recruiting
ABL501	Anti-PD1-LAG-3 bispecific antibody	Advanced solid tumors	1	NCT05101109	Recruiting
HLX26 HLX10	Anti-LAG-3 monoclonal antibody Anti-PD-1 monoclonal antibody	Adult solid tumor	1	NCT05400265	Not yet recruiting
HLX26	Anti-LAG-3 monoclonal antibody	Solid tumor Adult Lymphoma	1	NCT05078593	Recruiting
LBL-007 Toripalimab Axitinib	Anti-LAG-3 monoclonal antibody Anti-PD-1 monoclonal antibody Tyrosine kinase inhibitor	Advanced melanoma	1	NCT04640545	Recruiting
EMB-02	Anti-PD1-LAG-3 bispecific antibody	Advanced solid tumors	1/2	NCT04618393	Recruiting
FS118	Anti-PD1-LAG-3 bispecific antibody	Advanced cancer Metastatic cancer Squamous cell carcinoma of head and neck	1/2	NCT03440437	Recruiting
INCAGN02385 INCAGN02390 INCMGA00012	Anti-LAG-3 monoclonal antibody Anti-TIM-3 monoclonal antibody Anti-PD-1 monoclonal antibody	Melanoma	1/2	NCT04370704	Recruiting
Fianlimab (REGN3767) Cemiplimab Pembrolizumab Placebo	Anti-LAG-3 monoclonal antibody Anti-PD-1 monoclonal antibody Anti-PD-1 monoclonal antibody	Melanoma	3	NCT05352672	Recruiting

LAG-3: lymphocyte-activation gene 3; PD-1: programmed cell death protein 1; PD-L1: programmed cell death ligand 1; TIM-3: T-cell immunoglobulin and mucin domain 3

## New Anti-LAG-3/PD-1 Combinations

Apart from relatlimab, many molecules targeting LAG-3 are in clinical development (e.g., fianlimab (REGN3767) [NCT05352672], LAG525 [NCT03484923], MK4280 [NCT02720068], and RG6139 [NCT04140500]). In a phase 3 trial the efficacy and tolerability of fianlimab (REGN3767) in combination with cemiplimab compared to pembrolizumab in patients with previously untreated unresectable locally advanced or metastatic melanoma will be evaluated [NCT05352672]. Fianlimab is a newly developed fully human anti-LAG-3 antibody with high affinity, blocking LAG-3/MHC II driven T cell inhibition. Together with cemiplimab, a high affinity, human, hinge-stabilized IgG4 antibody to PD-1 receptor, fianlimab showed promising antitumor effects in preclinical studies. In a phase 1 dose escalation study [NCT03005782], combined therapy with fianlimab and cemiplimab demonstrated an acceptable safety profile and already indicated clinical activity in patients with advanced malignancies [54]. Preliminary data from two expansion cohorts of the phase 1 trial, in which anti-PD-1/PD-L1-naive and experienced patients with advanced melanoma were treated with fianlimab in combination with cemiplimab showed encouraging antitumor activity with an ORR of 63.8% for anti-PD-1/PD-L1-naive and 13.3% for anti-PD-1/PD-L1-experienced patients. DCR was 75.8% for anti–PD-1/PD-L1-naive patients and 40% for anti-PD-1/PD-L1 experienced patients and estimated PFS at 12 months follow-up was 60.6% for anti–PD-1/PD-L1-naive patients and 9.5% for anti–PD-1/PD-L1 experienced patients, whereas median PFS and median duration of response have not been reached for both cohorts. No correlation between LAG-3 or MHC II expression in immunohistochemistry and therapy response was found in either cohort and antitumor activity seemed to be independent of PD-L1 expression. LAG-3 inhibition was beneficial even in subgroups of poor prognosis e.g., elevated LDH levels or presence of liver metastasis [55•]. Notably, both patients with a complete response to dual therapy with fianlimab plus cemiplimab of the cohort of anti-PD-1/PD-L1 experienced patients had shown progressive disease as their best response to prior immunotherapy [56]. The combination of fianlimab and cemiplimab showed a similar safety profile to anti-PD-1 monotherapy except for adrenal insufficiency, which was slightly increased by 10.4%, and in turn, is comparable to the observed rate under dual checkpoint inhibition with anti-PD-1 and anti-CTLA-4. Treatment-related adverse events grade ≥ 3 occurred in 39.6% of patients, whereby 16.3% of patients discontinued therapy prematurely due to adverse events. Furthermore, numerous clinical trials are underway to investigate new anti-LAG-3 molecules in advanced solid tumors including melanoma (Table 3B).

## What Place Will the New Anti-LAG-3/anti-PD-1 Combo have in the Arsenal of Checkpoint Inhibitors?

The approval of the new combination therapy with relatlimab plus nivolumab for patients with advanced melanoma expands the arsenal of immune checkpoint inhibitors, raising new questions in clinical practice and requiring re-evaluation of currently established therapeutic standards and sequences. A key role is played by understanding factors that determine treatment response, resistance, and toxicity, in order to identify the appropriate combination and sequence of treatments for each patient and define an individually tailored therapy. Both, anti-PD-1 monotherapy or in combination with ipilimumab represent the current standard of care for patients with advanced melanoma. Although the Checkmate-067 trial was not powered enough to compare ipilimumab plus nivolumab with nivolumab alone, it demonstrated a numerical efficacy benefit [57•] establishing the combination as first-line therapy with a median PFS of 11.5 months after a minimum 60-month follow-up and a staggering median OS of 72.1 months [3]. Even though cross-trial comparisons should be made with caution, ipilimumab plus nivolumab and relatlimab plus nivolumab show comparable efficacy data with similar PFS rates (2-year PFS; 38.5 vs 43%) (Table 1) and OS rate (3-year OS; 55.8 vs 58%) [2, 8••, 34]. However, the high toxicity rate of ipilimumab and nivolumab with immune-mediated adverse events grade 3/4 in up to 59% of patients [2], requires its use to be carefully considered and the benefit-risk ratio to be weighed. First-line treatment may be moving towards the new anti-LAG-3/PD-1 combination due to its better safety profile. Yet, long-time survival data are lacking and data from the RELATIVITY-047 trial are still growing. It remains to be seen whether the new combination with relatlimab plus nivolumab will catch up to dual checkpoint inhibition with ipilimumab and nivolumab. It is unlikely that a large randomized head-to-head comparison of both combination treatments will be done as the differences in the safety profile are clearly in favor of the new combination and no significant difference in PFS or OS can be expected. More importantly, longer follow-up data are needed to clarify the difference in response between melanomas with high and low PD-L1 expression, especially since the EMA has approved combination therapy with relatlimab and nivolumab only for the treatment of melanomas with PD-L1 expression of less than 1%. The benefit of both ipilimumab plus nivolumab and relatlimab plus nivolumab was shown to be independent of key factors such as PD-L1 status, although both combinations showed their benefit, particularly in melanoma with PD-L1 expression of less than 1% (relatlimab/nivolumab vs Nivolumab: HR 0. 66 (95% CI, 0.51–0.84); ipilimumab/nivolumab vs nivolumab: HR 0.67 (95% CI, 0.51–0.84) [4, 8••]. However, it could have been demonstrated that the tumor-derived PD-L1 expression was not predictive of response to ipilimumab and nivolumab therapy [3]. Longer follow-up data are needed to shed light on the difference in response between melanomas with high and low PD-L1 expression under therapy with relatlimab plus nivolumab.

Although the new dual checkpoint inhibition shows positive antitumor activity and appears to be effective by prolonging PFS, a large proportion of patients will not respond to therapy or will experience disease progression after a period of response, requiring follow-up therapy. To date, we do not know whether the same patients who do not respond to relatlimab plus nivolumab also do not respond to ipilimumab plus nivolumab or vice versa and whether they differ. In a small, pooled, retrospective, multicenter analysis, anti-CTLA-4-based therapy was shown to be less effective after treatment failure on relatlimab plus nivolumab with an overall response rate of 11%, although the cohort was too small to allow robust analysis between ipilimumab mono and combined therapy with ipilimumab plus nivolumab [58•]. In the updated results of Part D of the RELATIVITY-020, an open-label Phase I/II study, the combination of relatlimab plus nivolumab demonstrated similar clinical activity, albeit lower than the first-line setting, in patients with advanced melanoma who had previously failed to respond to one or more anti-PD-1 or anti-PD-L1-containing therapies, with ORRs of 12.0% and 9.2%, respectively *(unpublished data)* [59••]. Within the C-144–01 study, which evaluates the efficacy and safety of Lifileucel, an investigational autologous tumor-infiltrating lymphocyte (TIL) cell therapy in patients with advanced melanoma previously treated with anti-LAG3 antibody a small proportion of patients who failed prior therapy with nivolumab plus relatlimab achieved durable responses with an ORR of 38.5% [60].

Furthermore, it remains to be seen what value the new dual checkpoint combination will have in distinct patient groups such as brain metastases, elevated LDH and liver metastases. Therapy with ipilimumab and nivolumab shows superior efficacy with long-lasting effects compared with anti-PD-1 monotherapy while inducing both extracranial and intracranial responses. Based on the results of two phase 2 trials the combination therapy with ipilimumab and nivolumab lead to an ORR of up to 56% intracranially in patients with active brain metastases. Thus, it has become established as first-line treatment in melanoma patients with brain metastases [61, 62]. Since the RELATIVITY-047 study included only 2% of patients with treated and asymptomatic brain metastasis, it is still unclear whether dual checkpoint inhibition with nivolumab plus relatlimab will have similar effects intracranially [8••].

## Conclusions

The need for oncological treatment options with an improved benefit-to-risk ratio has increased with new treatment options for patients that can significantly prolong survival and, in some cases, lead to cure. Dual immune checkpoint inhibition has become the focus of research by both prolonging duration of response and improving therapy response rates [63]. Relatlimab is the third immune checkpoint inhibitor to receive approval for advanced melanoma therapy, along with ipilimumab and nivolumab/pembrolizumab, and has shown significant improvement in PFS survival in therapy-naive advanced melanoma compared with anti-PD-1 monotherapy when used in combination with nivolumab [8••]. PFS and 3-year OS for both combination immunotherapies are similar, the safety profile of relatlimab plus nivolumab appears to be more favorable than that of ipilimumab plus nivolumab. Neither relatlimab plus nivolumab nor ipilimumab plus nivolumab could demonstrate a significant OS benefit versus nivolumab monotherapy [8••, 19]. Ultimately, apart from long-term data for relatlimab plus nivolumab, predictive, and prognostic biomarkers and risk prediction tools that include patient- and tumor-related clinical factors are needed to determine the appropriate treatment combination and sequence for each patient.
